# Insights into the Complexity of a Dormant *Mycobacterium tuberculosis* Cluster Once Transmission Is Resumed

**DOI:** 10.1128/spectrum.01381-21

**Published:** 2022-01-19

**Authors:** Fermin Acosta, Miguel Martínez-Lirola, Pedro J. Sola-Campoy, Jon Sicilia, Teresa Guerra-Galán, Sandra R. Maus, Patricia Muñoz, Laura Pérez-Lago, Darío García de Viedma

**Affiliations:** a Servicio de Microbiología Clínica y Enfermedades Infecciosas, Hospital General Universitario Gregorio Marañón, Madrid, Spain; b Instituto de Investigación Sanitaria Gregorio Marañón, Madrid, Spain; c Complejo Hospitalario Torrecárdenas, Almería, Spain; d CIBER Enfermedades Respiratorias (CIBERES), Madrid, Spain; e Departamento de Medicina, Universidad Complutense, Madrid, Spain; Houston Methodist Hospital

**Keywords:** tuberculosis, cluster, transmission, reactivation, within-host diversity, clonal complexity, *Mycobacterium tuberculosis*

## Abstract

Genotyping tools help identify the complexity in Mycobacterium tuberculosis transmission clusters. We carried out a thorough analysis of the epidemiological and bacteriological complexity of a cluster in Almería, Spain. The cluster, initially associated with Moroccan migrants and with no secondary cases identified in 4 years, then reappeared in Spanish-born individuals. In one case, two Mycobacterium tuberculosis clonal variants were identified. We reanalyzed the cluster, supported by the characterization of multiple cultured isolates and respiratory specimens, whole-genome sequencing, and epidemiological case interviews. Our findings showed that the cluster, which was initially thought to have restarted activity with just a single case harboring a small degree of within-host diversity, was in fact currently growing due to coincidental reactivation of past exposures, with clonal diversity transmitted throughout the cluster. In one case, within-host diversity was amplified, probably due to prolonged diagnostic delay.

**IMPORTANCE** The precise study of the dynamics of tuberculosis transmission in socio-epidemiologically complex scenarios may require more thorough analysis than the standard molecular epidemiology strategies. Our study illustrates the epidemiological and bacteriological complexity present in a transmission cluster in a challenging epidemiological setting with a high proportion of migrant cases. The combination of whole-genome sequencing, refined and refocused epidemiological interviews, and in-depth analysis of the bacterial composition of sputa and cultured isolates was crucial in order to correctly reinterpret the true nature of this cluster. Our global approach allowed us to reinterpret correctly the unnoticed epidemiological and bacteriological complexity involved in the Mycobacterium tuberculosis transmission event under study, which had been overlooked by the usual molecular epidemiology approaches.

## INTRODUCTION

Molecular epidemiology strategies have been instrumental in detecting tuberculosis transmission. These strategies are based on identifying clusters of cases infected by the same strain, which are used as a proxy for recent transmission (1).

Cluster surveillance in control programs, in which all Mycobacterium tuberculosis isolates from whole populations are systematically genotyped, can help identify those strains responsible for major clusters that are most actively transmitted and causing new secondary cases, so that targeted control measures can be applied. It is essential to differentiate such clusters from those that do not comprise new secondary cases, which can be considered under control. Accuracy in differentiating recent transmission cases from others has increased with the advent of genomic epidemiology (2–5).

The systematic use of genotyping tools for epidemiological purposes has provided relevant information on within-host diversity in M. tuberculosis infections, especially mixed infections involving more than one strain or the coexistence of clonal variants emerging from a parental strain as a result of microevolution events (6, 7). Potential within-host diversity in M. tuberculosis infections is generally not considered in transmission surveillance programs, which limits accuracy when defining clusters.

Here, we carried out a thorough analysis of an epidemiologically and bacteriologically complex cluster in Spain. The cluster strain, whose transmission seemed to be under control for several years, was initially linked to Moroccan migrants. Unexpectedly, new clustered cases involving only Spaniards were identified. Whole-genome sequencing (WGS) analysis, together with refocused epidemiological interviews, enabled us to understand what led to reactivation of the cluster. We also observed that several clonal variants had been transmitted between the cluster cases but had been missed due to their differential ability to be recovered in culture. Finally, possibly due to a delay in diagnosis, greater within-host diversity and recovery of the ability of one variant to be cultured were observed in one of the latest cases.

## RESULTS

Between 2009 and 2012, we identified an active mycobacterial interspersed repetitive unit-variable number tandem repeat (MIRU-VNTR)-defined cluster (cluster 1192; Table 1) in a molecular epidemiology program in Almería (8), southeast Spain. The cluster involved seven cases (pansusceptible strain, lineage 4; LAM). All but two of the cluster cases involved Moroccan migrants, including the last case added in 2012; the remaining two involved one Romanian individual and one Spanish individual. No cases linked to this cluster were identified by the systematic population-based molecular epidemiology program in the following 4 years (2013 to 2016). This suggested that the chain of transmission associated with this cluster was under control. Between 2017 and 2018, however, three Spanish-born cases, infected with the same strain (based on VNTR data), were detected (Table 1).

**TABLE 1:** MIRU-VNTR patterns of isolates in cluster 1192^*a*^

Case	Origin	Yr	MIRU-VNTR typing																								Source or reference
			M02	M20	M23	M24	M27	M39	M4	M26	M40	M10	M16	M31	M42	M43	ETRA	47	52	53	Qub11b	1995	Qub26	M46	M48	M49	
1192	Morocco	2009	1	2	6	1	3	2	2	5	4	4	2	3	3	4	2	1	2	3	2	3	6	4	2	3	8
1194	Morocco	2009	1	2	6	1	3	2	2	5	4	4	2	3	3	4	2	1	2	3	2	3	6	4	2	3	8
1360	Morocco	2010	1	2	6	1	3	2	2	5	4	4	2	3	3	4	2	1	2	3	2	3	6	4	2	3	8
1409	Romania	2011	1	2	6	1	3	2	2	5	4	4	2	3	3	4	2	1	2	3	2	3	6	4	2	3	8
1505	Morocco	2011	1	2	6	1	3	2	2	5	4	4	2	3	3	4	2	1	2	3	2	3	6	4	2	3	8
1527	Spain	2011	1	2	6	1	3	2	2	5	4	4	2	3	3	4	2	1	2	3	2	3	6	4	2	3	8
1545	Morocco	2012	1	2	6	1	3	2	2	5	4	4	2	3	3	4	2	1	2	3	2	3	6	4	2	3	8
2275	Spain	2017	1	2	6	1	3	2	2	5	4	4	2	3	3	4	2	1	2	3	2	3	6	4	2	3	This study
2398	Spain	2018	1	2	6	1	3	2	2	5	4	4	2	3	3	4	2	1	2	0/3	2	3	6	4	2	3	This study
2443	Spain	2018	1	2	6	1	3	2	2	5	4	4	2	3	3	4	2	1	2	3	2	3	6	4	2	3	This study

a The case numbers, countries of origin, and years of diagnosis are shown. The new cases in this study and historical cases from a previous report are indicated.

### Epidemiological complexity.

Our initial interpretation was that cluster 1192 had reactivated after being epidemiologically dormant for 4 years. At the same time, the transmission pattern of the cluster had changed from one mostly associated with Moroccan migrants to one involving only Spanish individuals.

We searched for evidence of recent transmission responsible for the infection of the three new Spanish cases and focused on interviewing case 2443, who was employed in a coffee shop. At this point, we found no relationships between the cases.

**FIG 1:**
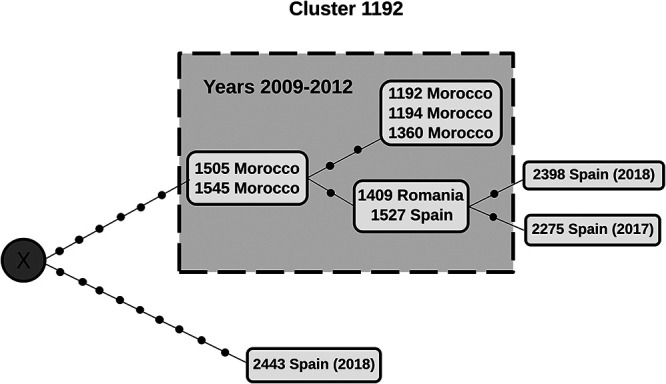
Network of genomic relationships generated from whole-genome sequencing analysis of cultured isolates from cluster 1192 cases. The dashed box contains cluster cases for the years 2009 to 2012. The three cases identified after the cluster resumed activity are represented outside the box. Each black dot represents a single nucleotide polymorphism. When two or more cases share an identical genome (zero single nucleotide polymorphisms between them), they are included in the same box. X, not sampled recent common ancestor for the two branches.

WGS data were available (8) for 6 of the 7 cases identified between 2009 and 2012. To complete the genomic network for cluster 1192, we performed WGS analyses of isolates from the four newly identified cases. WGS results for the new cases showed that there was no direct relationship between these cases (Fig. 1), although cases 2398 and 2275 were genetically related to cases diagnosed in 2011 (1527 Spanish-born and 1409 Romanian; see Fig. 1) but not to each other. Each had a single unique single-nucleotide polymorphism (SNP) that positioned them in different branches of the network (Fig. 1). Meanwhile, case 2443, the last case to be identified (year 2018) as part of the cluster, was completely unrelated to cases 2398 and 2275 or to any other member of cluster 1192. Nine SNPs unique to case 2443 were identified. All other members of cluster 1192 shared six SNPs not shared by case 2443 (Fig. 1). In other words, there was an unsampled ancestor within the network, located at the hub of the two branches of transmission, one involving all the cases in the cluster except one, and the other represented by case 2443.

This information indicated that our initial assumption of a new, currently active chain of transmission involving Spaniards was wrong and that reactivation of past exposures was the most likely hypothesis. Especially informative was the positioning of the last Spanish case (i.e., case 2443, year 2018) in the network of genomic relationships (Fig. 1): distant from the group and closer to the unsampled ancestor common to the first Moroccan cases in the cluster. When case 2443 was interviewed again, she was questioned about possible past relationships with Moroccan individuals. Case 2443 remembered having close contact more than 10 years previously with a Moroccan migrant who was employed on a cattle farm she owned. That employee had been ill at the time, with symptoms compatible with tuberculosis. It was not possible to reinterview the Moroccan case since he had returned to Morocco in search of medical care after developing symptoms.

### Microbiological complexity: analysis of cultured isolates.

**FIG 2:**
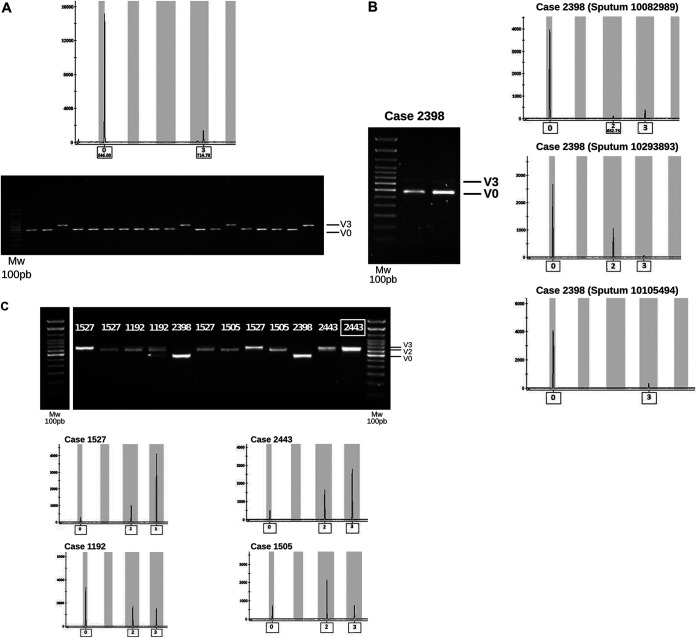
(A) VNTR53 locus amplicons from one isolate from case 2398 (top panel) and a representative selection of single colonies from an isolate from case 2398 (bottom panel). (B and C) Segregated variants with alleles 0 and 3 at locus VNTR 53 are included as controls (white arrows): direct analysis of case 2398 sputa by standard (left panel) and capillary electrophoresis (right panel) (B) and sputa from a selection of cases from the cluster (numbers indicate the case reference number) by standard (top panel) and capillary electrophoresis (central and bottom panels) (C). Numbers in squares indicate a reference allele control obtained from a cultured isolate.

One of the new members of the cluster caught our attention. In case 2398, a single locus variant (SLV; allele 0) was detected at locus VNTR 53, in addition to allele 3, shared by all the other cases in the cluster (Table 1). The allelic intensity of allele 0 in case 2398 was higher than that of the shared allele 3 (Fig. 2A). The case was a 45-year-old, HIV-negative female with prolonged diagnostic delay and symptoms compatible with tuberculosis (TB) over the preceding 4 years. At diagnosis, she presented cavitary lesions and a high bacillary load.

Accordingly, we confirmed that the presence of the double allele corresponded to two coinfecting variants. Analysis of 40 single colonies allowed us to segregate each variant (Fig. 2A) and determine the predominance of variant 0 over variant 3 (9:1 ratio). This was consistent with the higher intensity of allele 0 versus allele 3 in our first observations obtained directly from cultures (Fig. 2A).

We also sought to clarify whether the within-host diversity observed in case 2398 had emerged in that patient or was present in other previous cluster cases. A careful review of the electropherograms confirmed that no other case had variant 0 and that only variant 3 was present.

### Direct analysis on respiratory specimens: standard genotyping.

We then set out to test whether the relative proportion of the two variants identified in the cultured isolate from case 2398 corresponded to the true composition of the bacterial population infecting the patient. To do this, we performed direct analyses of the sputum. The intensity of the amplicons confirmed the predominance of variant 0 (Fig. 2B). A new variant (allele 2) was detected in two of the three sputum samples analyzed. Analyses of 40 colonies obtained after directly plating the sputa showed the same 9:1 proportion of variants 0 and 3 observed in the cultured isolate. Variant 2 was not identified in single colony analyses.

To assess whether within-host diversity had been overlooked in the other patients in the cluster when analyzing cultured isolates, we directly examined sputum samples from another four cases, including the first case in the cluster (case 1192) and the one preceding case 2398 in the genomic network (case 1527) (Fig. 1); there were two sputa from case 1192; two from case 1505, four from case 1527, and one sputum from case 2443 (Table 2). Variants 0, 2, and 3 were detected in all cases, although with various degrees of representativeness among the sputa analyzed (Table 2 and Fig. 2C). In every case but one, variant 0 was much less represented than the other variants (Fig. 2C).

**TABLE 2:** Alleles obtained for locus VNTR 53 from direct analysis of sputa by analyzing amplicon sizes by capillary electrophoresis^*a*^

Case	Sputum	Capillary	Bacillary load (200×)	Sputum collection date
1192	23885	2/3	>90 AFB/field	2009-12-22
1192	23685	0/2/3	>90 AFB/field	2009-12-21
1505	86341	0/2/3	1–9 AFB/field	2011-11-07
1505	86323	2/3	1–9 AFB/10 fields	2011-11-07
1527	93978	2/3	10–90 AFB/field	2012-01-04
1527	94085	2/3	>90 AFB/field	2019-01-05
1527	93896	0/2/3	>90 AFB/field	2012-01-03
1527	93627	0/2/3	>90 AFB/field	2011-12-30
2443	10336392	0/2/3	10–90 AFB/field	2018-10-19
2398	10082989	0/2/3	>90 AFB/field	2018-06-22
2398	10293893	0/2/3	10–90 AFB/field	2018-08-14
2398	10105494	0/3	>90 AFB/field	2018-06-22

a Bacillary loads and collection dates of sputa are indicated. AFB, acid-fast bacilli.

For single colony analysis, plating was done using two and three sputa, respectively, for cases 1192 and 1527. Forty colonies were analyzed from each sputum from case 1192, and 76, 33, and 18 colonies from the sputum samples from case 1527. Variant 2 corresponded to just one colony from patient 1527, and variant 3 was identified in all remaining colonies for both cases.

Direct genotyping on the respiratory samples and single colonies obtained from these specimens revealed that three variants were infecting several cases and had been transmitted throughout the cluster. This information had been overlooked in the analyses of cultured isolates (two of these variants were not recovered after culturing). Only in case 2398 did one of these variants appear to have recovered the ability to be cultured.

**FIG 3:**
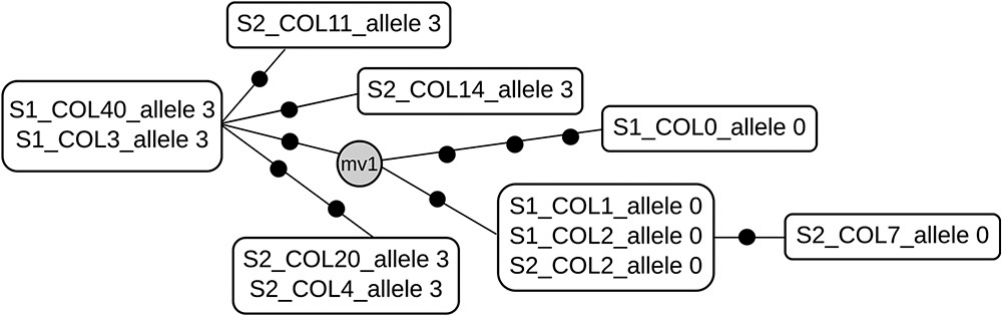
Network of genomic relationships generated from WGS data in single-colony analyses from case 2398. Each black dot represents an SNP. When two or more cases share an identical genome (zero SNPs between them), they are included in the same box. The allele corresponding to the VNTR 53 locus is shown. S, sputa; COL N, colony number; mv1, median vector corresponding to a node not occupied by any of the sequenced specimens (unsampled node).

A selection of single colonies from directly plated sputa were also analyzed by WGS: six colonies from case 1192 (variant 3), eight colonies from case 1527 (one variant 2, the remainder variant 3), and 11 colonies from two sputa from case 2398 (six from variant 3 and five from variant 0). SNP-based diversity was not detected among the colonies for case 1192. Similarly, all colonies corresponding to variant 3 in case 1527 were identical, with only minimal diversity (1 SNP) detected for the colony representing variant 2. In case 2398, however, greater diversity was found among colonies, variants (six SNPs) and colonies representing the same variant (3 to 4 different genotypes, differing by 1 to 5 SNPs) (Fig. 3).

## DISCUSSION

Molecular epidemiology strategies have limitations when used to determine chains of recent tuberculosis transmission in complex socio-epidemiological settings. In a cluster linked primarily to Moroccan migrants, we previously demonstrated that the greater discriminatory power of a WGS-based analysis was able to differentiate true cases of recent transmission from other overlapping phenomena, such as independent importations of variants sharing the same MIRU-VNTR pattern (8). The limitations of MIRU-VNTR typing for identifying cases due to recent transmission involving migrants or asylum seekers have also been found in other settings (2, 9).

The use of WGS in the MIRU-VNTR-based cluster analyzed in this study increased our understanding of the reasons for the sudden appearance of cases associated with a cluster that had stayed been inactive for several years. The distribution of cases, based on SNPs, ruled out the suspicion of a new focus of active transmission. Instead, the coincidental reactivation of exposures, which had probably occurred when the cluster was active, seemed to be the most likely explanation. This new interpretation is partly supported by the new links identified during epidemiological interviews with affected subjects. Our results are in line with other studies, in which the combined effect of the refined data provided by WGS and the epidemiological information obtained from detailed interviews, which were oriented based on the distribution of cases in the networks of SNPs, were essential to uncover the true causes of transmission in clusters with no obvious epidemiological links (10, 11).

Besides addressing the epidemiological complexity in cluster 1192, we also dissected clonal diversity within the cluster. In other studies across multiple cases over time, diversity within clusters is not observed (4, 12). While we acknowledge that not all sputa/isolates from our cluster under study were available, nevertheless, the clonal diversity described within it was remarkable, despite this limitation. The first observation of within-host diversity in this cluster, in which two clonally related VNTR variants were identified, involved only one of the recently diagnosed cases. This within-host diversity, based on a SLV (at VNTR 53, alleles 0/3), was revealed with MIRU-VNTR, a genotyping tool with intermediate discriminatory power. In one large nationwide population-based study, this type of complexity involving related variants (SLVs) detected by MIRU-VNTR was estimated to be 2.2% (13), and there have been estimates of 1.2% and 5% in other studies focused on local populations in Brussels (14) and Vietnam (15), respectively.

Most of the attempts to estimate the magnitude of clonally complex infections in tuberculosis have been based on analysis of cultured isolates. If we had limited our study to this standard strategy, we would have interpreted our findings as the emergence of clonal complexity in a single case in the cluster, probably due to diagnostic delay. Instead, by extending our characterization to the direct analysis of respiratory specimens, we were able to identify clonally related variants across the cluster that preceded the case initially detected as complex. This finding suggests the transmission of these variants along the cluster. A limitation of our study is that it is difficult to ascertain from our data whether the initial case was already coinfected with related clonal variants or whether within-host microevolution occurred after infection and the clonal variants were transmitted subsequently. There are only sporadic reports of transmission of more than one variant within a transmission cluster. In the largest study performed in the Netherlands, two variants were transmitted to at least one additional case in 13% of clusters with variants, and both variants were transmitted to all clustered cases in just one of 86 clusters (13).

It is worth noting that, in most cases, the diversity was detected exclusively by direct analysis of respiratory specimens and not from cultured isolates. It has been shown that culture can affect the composition of *M. tuberculosis* populations in sputum (16). The lack of diversity from cultured samples has been reported at the level of macrodiversity; some lineages grow poorly in culture and may therefore be difficult to detect (17, 18). Furthermore, lower within-sample diversity has been described at the level of microdiversity, based on the reduction in heterozygous alleles when analyzing cultured isolates versus the original specimens (19).

In addition to showing the importance of using sputa to determine the true diversity in the cluster under study, we also note that it is not always comparable between sputa. It has been suggested that each sputum may represent only part of the true complexity in the lung, in other words, that each sputum captures the bacterial composition of lesions draining bacilli at that specific sampling time, so that it is necessary either to analyze several sequential sputa to identify the different variants infecting a patient or to collect samples from the lower respiratory tract (20). Finally, at the level of microdiversity, different variants with different SNPs can be found in different cavitary lesions and thus are not systematically detected in each sputum specimen (21).

Once we determined that diversity was being transmitted throughout the cluster, we noted that it was most marked in the case in which within-host diversity was initially detected. This case was the only one in the cluster to show diversity when cultured isolates were analyzed, without the need for a deeper direct analysis on respiratory specimens, while the greatest diversity, as revealed by WGS, was also seen in this case. It is highly likely that prolonged diagnostic delay in this case led to higher diversity and the recovery of fitness by variant 0 (which was detected after culturing only in this case). Within-host diversity has been found more frequently in older subjects and drug users, the latter probably linked to diagnostic delays (13).

The diversity described here consists of subtle changes that may be interpreted as irrelevant. However, it has been demonstrated that minimally different variants show differences when infecting macrophages, as well as in mouse models (22, 23). The subtle changes acquired in our case may have selected for a fitter variant. Unfortunately, it was not possible to recover variant 0 from other cases and compare their WGS data to identify the potential SNPs involved.

Despite the limitation that our observations were obtained from just a single cluster, our study illustrates the epidemiological and bacteriological complexity present in the cluster under study. The combination of WGS, refined and refocused epidemiological interviews, in-depth analysis of the bacterial composition of sputa, and cultured isolates was key to correctly reinterpret the true nature of this cluster. In summary, the results presented here show that (i) a cluster initially considered to have restarted activity due to a transmission hot spot, with a single case harboring a small degree of within-host diversity, was in fact (ii) a cluster that was currently growing as a result of coincidental reactivations of past exposures, with clonal diversity transmitted throughout the cluster, including a case in which this diversity was amplified, causing a functional impact on fitness, probably due to a prolonged diagnostic delay.

## MATERIALS AND METHODS

DNA was purified from subcultures in Mycobacterium growth indicator tubes (MGIT; Becton Dickinson, Franklin Lakes, NJ) or from sputa, using a Qiagen kit (QIAamp DNA minikit; Qiagen, Courtaboeuf, France). For individual colony analysis, we performed serial dilutions of a concentrated inoculum (0.5-0.8 McFarland) and plated the broth dilutions (10^2^ to 10^6^) on 7H11 agar plates. When the total number of colonies obtained was <40, all were selected, and when the number exceeded 40, we took a sector in the plate with ∼40 colonies and selected all of them. Boiled crude extracts from the colony suspended in phosphate-buffered saline were used for genotyping.

### MIRU-VNTR typing.

MIRU-VNTR analysis was carried out with a multiplex PCR kit (Qiagen) according to a standard procedure (24). Sizing of PCR fragments was by capillary electrophoresis (3130 genetic analyzer; Applied Biosystems, Foster City, CA). For MIRU-VNTR allele assignment, we used GeneMapper 4.0 software (Thermo Scientific, Waltham, MA). The presence of alternative alleles at a single locus was determined by running the amplification products on standard agarose gels.

MIRU-VNTR clusters were defined for isolates sharing an identical pattern at all 24 loci analyzed. Within-host diversity in a cluster was considered when double alleles were detected in at least one MIRU-VNTR locus in one cluster isolate.

### Whole-genome sequencing.

Libraries were prepared using the Nextera XT kit (Illumina, San Diego, CA) and run in the MiSeq 2000 system. Sequence fastq files were deposited in the European Nucleotide Archive (http://www.ebi.ac.uk/ena; see accession number PRJEB25814 for sequences from our previous report [8] and PRJEB42555 and PRJEB48947 for the sequences from this study).

Quality filtering and coverage calculation were done prior to variant calling using a modified in-house analysis pipeline (https://github.com/MG-IiSGM/covid_multianalysis). Sequences with at least 20× coverage were filtered using BBtools v38.73 (https://sourceforge.net/projects/bbmap/) and then mapped against a reference genome (the most recent common ancestor of the *M. tuberculosis* complex) (25) with the Burrows-Wheeler Aligner package v0.7.17 (26). GATK Best Practices were followed for variant calling (Genome Analysis Tool Kit) v4.0.5.1 (27). Only calls with frequencies over 80% and properly called (mean SNP mapping quality of 20) in at least 90% of the samples were included. Highly polymorphic and repetitive regions, phages, and PE/PPE regions were removed from the final SNP distance calculation and annotation (28). SNPs located close to indels or in areas with a higher-than-expected number of calls (≥3 SNPs within 10 bp of each other) were also excluded.

Alignments and SNP variants were visualized and checked with the IGV (Integrative Genomics Viewer) program (https://software.broadinstitute.org/software/igv/AlignmentData). Median-joining networks of genomic relationships were constructed from the SNP matrix generated using NETWORK 5.0.0.1. Median vectors were defined when the distribution of SNPs indicated the existence of a nonsequenced node corresponding to a genotype that had not been sampled in the cluster. The chronology of SNP acquisition is represented from left to right in the networks.

### Data availability.

The data that support the findings of this study (FastQ files) are openly available. Sequence Fastq files have been deposited in the European Nucleotide Archive (http://www.ebi.ac.uk; accession number PRJEB42555).
