# Erratum for Acosta et al., “Insights into the Complexity of a Dormant Mycobacterium tuberculosis Cluster Once Transmission Is Resumed”

**DOI:** 10.1128/spectrum.02544-22

**Published:** 2022-08-15

**Authors:** Fermin Acosta, Miguel Martínez-Lirola, Pedro J. Sola-Campoy, Jon Sicilia, Teresa Guerra-Galán, Sandra R. Maus, Patricia Muñoz, Laura Pérez-Lago, Darío García de Viedma

**Affiliations:** a Servicio de Microbiología Clínica y Enfermedades Infecciosas, Hospital General Universitario Gregorio Marañón, Madrid, Spain; b Instituto de Investigación Sanitaria Gregorio Marañón, Madrid, Spain; c Complejo Hospitalario Torrecárdenas, Almería, Spain; d CIBER Enfermedades Respiratorias (CIBERES), Madrid, Spain; e Departamento de Medicina, Universidad Complutense, Madrid, Spain

## ERRATUM

Volume 10, no. 1, e01381-21, 2022, https://doi.org/10.1128/Spectrum.01381-21. In the Acknowledgments, lines 2 to 4 should read as follows: “This study was supported by the ERANet-LAC (TRANS-TB-TRANS Project ELAC2015/T08-0664) and the ISCIII (AC16/00057, 15/01554, 16/01449, 18/00599, 19/00331, and contract CPII20/00001 for L.P-L.), cofunded by the European Regional Development Fund (ERDF: “A way to make Europe”) from the European Commission and the Junta de Andalucía (PI0488-2017). . . .”

